# A new finger-preserving procedure as an alternative to amputation in recurrent severe Dupuytren contracture of the small finger

**DOI:** 10.1186/s12891-019-2701-2

**Published:** 2019-07-09

**Authors:** Asa Eiriksdottir, Isam Atroshi

**Affiliations:** 1 Department of Orthopedics, Hässleholm-Kristianstad Hospitals, SE-28125 Hässleholm, Sweden; 2 Department of Clinical Sciences – Orthopedics, Lund University, SE-22100 Lund, Sweden

**Keywords:** Dupuytren disease, Amputation, Middle-phalanx excision

## Abstract

**Background:**

Recurrent severe Dupuytren contracture of the small finger’s proximal interphalangeal (PIP) joint is a difficult problem. Further surgery carries high risk of complications and poor outcome. Patients are often offered finger amputation. We have devised a novel surgical procedure consisting of middle phalanx monoblock resection and ligament reconstruction to create a new functioning interphalangeal joint.

**Methods:**

Two patients requesting small-finger amputation because of severe PIP joint contracture after multiple treatments for Dupuytren contracture were offered and accepted this new procedure. Through a dorsal incision the extensor tendon is incised longitudinally exposing the middle phalanx and interphalangeal joints. The collateral ligaments of both interphalangeal joints are detached from the middle phalanx. The middle phalanx is dissected from soft tissues (including the flexor digitorum superficialis tendon) and removed. The distal phalanx is brought proximally and the ends of the collateral ligaments are sutured with non-absorbable sutures with the joint held in full extension and congruency. The two patients were evaluated at 18 months and 15 months after surgery, respectively.

**Results:**

Both patients regained good finger posture with almost full extension and had normal sensation and no pain. Active flexion in the new interphalangeal joint was 60 degrees and 35 degrees, respectively. Both patients had full metacarpophalangeal joint flexion and extension, normal 2-point discrimination in the small finger and higher grip strength in the treated than the contralateral hand. Radiographs showed a congruent new interphalangeal joint. Both patients were very satisfied with the outcome.

**Conclusions:**

In patients with Dupuytren disease and severe PIP joint contracture after multiple treatments, this novel procedure consisting of middle-phalanx excision and ligament reconstruction creating a new functioning interphalangeal joint has good short-term outcomes and is a favorable alternative to finger amputation. Longer follow-up will show whether these results are durable.

## Background

In patients with Dupuytren disease (DD) recurrent contracture of the proximal interphalangeal (PIP) joint of the small finger is a difficult problem. The severely contracted finger impedes activities of daily living and work. Various treatments have been proposed including traction by external fixator followed by surgery [1, 2], staged surgery [3], amputation with ray resection [4], and reconstruction using local skin flaps [5, 6]. Other more complex procedures involve complete skin and fascial excision and resurfacing with regional tissue transfer such as forearm-based flap and possible free flap (adipofascial radial forearm flap or free temporoparietal fascia flap). However, results in terms of residual symptoms, hand function and cosmetic appearance have generally been unsatisfactory.

When all surgical and other treatments have failed the only remaining option to offer patients has often been amputation of the affected finger. It has been estimated that finger amputations constitute approximately 2% of all surgical procedures performed on patients with DD [7, 8]. The small finger is the most common finger on which amputation is performed. Amputation is associated with risks such as phantom pain and cold intolerance that, if severe, may substantially affect patient’s quality of life [9–12]. In addition, the cosmetic result of finger amputation is troublesome for some patients.

As an alternative to finger amputation we have devised a novel surgical procedure that involves total excision of the middle phalanx and creation of a functioning single interphalangeal joint. To our knowledge, this procedure has not been described previously although the underlying concept, removal of the middle phalanx, has been reported in 2 previous publications. Honecker et al. described a procedure in which the middle phalanx of the small finger is removed followed by fusion of the proximal and distal phalanx in 7 patients with DD or posttraumatic PIP joint contracture [13]. Teboul et al. has described a procedure, done on a patient with recurrent Dupuytren contracture of the small finger after two previous subtotal fasciectomy procedures, involving simple removal of the middle phalanx but without the ligament reconstruction necessary to create a functioning interphalangeal joint [14].

We have performed this novel finger-preserving procedure of total excision of the middle phalanx and creation of a functioning single interphalangeal joint on two patients with recurrent severe Dupuytren contracture of the small finger.

## Methods

This procedure was offered and carried out as an alternative to amputation on two patients who presented with severe contracture of the small-finger PIP joint after multiple treatments for DD.

Patient 1 was a 56 years old man with bilateral DD who presented with severe contracture of the PIP joint of the small finger in his left hand after previous fasciectomy and collagenase injection (Table 1). The patient was a non-smoker with diabetes and a previous history of Guillan-Barré syndrome. After the previous treatments for DD the contracture recurred within few months and worsened rapidly. The patient requested finger amputation but accepted to undergo this procedure with the knowledge that the surgeon during the course of surgery, might decide to perform finger amputation.

**Table 1:** Patient characteristics and outcome

	Patient 1	Patient 2
Age (y)	56	70
Sex	Male	Male
Treated hand	Left	Right
Preoperative passive extension deficit (degrees)		
Proximal interphalangeal joint	80	70
Distal interphalangeal joint	60	0
Follow-up time (months)	18	15
Postoperative range of motion (degrees)		
New interphalangeal joint		
Active	20–60	10–35
Passive	15–65	0–40
Metacarpophalangeal joint, active	10–90	0–90
Grip strength, treated: contralateral (kg)	50: 45	27: 21
Two-point discrimination, radial: ulnar (mm)	5: 5	5: 5

Patient 2 was a 70 years old man with bilateral DD. He was a smoker and had hypertension but no other medical co-morbidities. He presented with severe contracture of the right hand small-finger PIP joint (Table 1). He had previously undergone surgical fasciectomy 3 times and received collagenase injection. He requested finger amputation because of debilitating symptoms but was offered this procedure as an alternative.

### Surgical procedure

The procedure was carried out under regional anesthesia and tourniquet. A longitudinal dorsal incision was used in the first patient and a curved incision (apex at the PIP joint) in the second patient. The curved incision was found to better facilitate partial excision of redundant skin. Dorsal veins are preserved to avoid potential venous congestion. The extensor tendon was incised longitudinally. The middle phalanx together with the PIP and distal interphalangeal (DIP) joints were exposed. The soft tissues containing the neurovascular bundles were protected. The joint capsules of both interphalangeal joints were opened and the collateral ligaments identified. The collateral ligaments of the PIP joint were released from their distal attachment and those of the DIP joint were released from their proximal attachment and protected for later repair. The middle phalanx was excised by releasing it from the surrounding soft tissues including the volar structures and removed en bloc. This involved incision of the volar plates of the PIP and DIP joints and releasing the FDS and allowing it to retract. The distal phalanx was brought to the proximal phalanx and the ends of the collateral ligaments were sutured together with non-absorbable sutures with the joint held in full extension and congruency. The joint capsule was closed on the dorsal side; the remaining parts of the volar plates were left in situ. The longitudinally incised extensor tendon (including the released central slip and the lateral bands still attached to the distal phalanx) was sutured with non-absorbable sutures with plication to obtain appropriate tension. All volar structures were left untouched. The skin was closed with nonabsorbent sutures. A soft dressing and a plaster splint was applied.

### Postoperative care

The dressing was changed 1 week postoperatively and the new interphalangeal joint was immobilized with a splint in full extension. The patients started hand therapy with active range of motion exercises of the metacarpophalangeal (MCP) joints. At 2 weeks the sutures were removed. At 4 weeks after surgery active motion of the new interphalangeal joint was initiated with buddy taping.

### Follow-up examination

The two patients were evaluated at 18 months and 15 months after surgery, respectively. The patients attended physical examination including measurements of range of motion with a handheld goniometer, grip strength with the Jamar Dynamometer, and 2-point discrimination. They also underwent radiological examination.

## Results

**Fig. 1:**
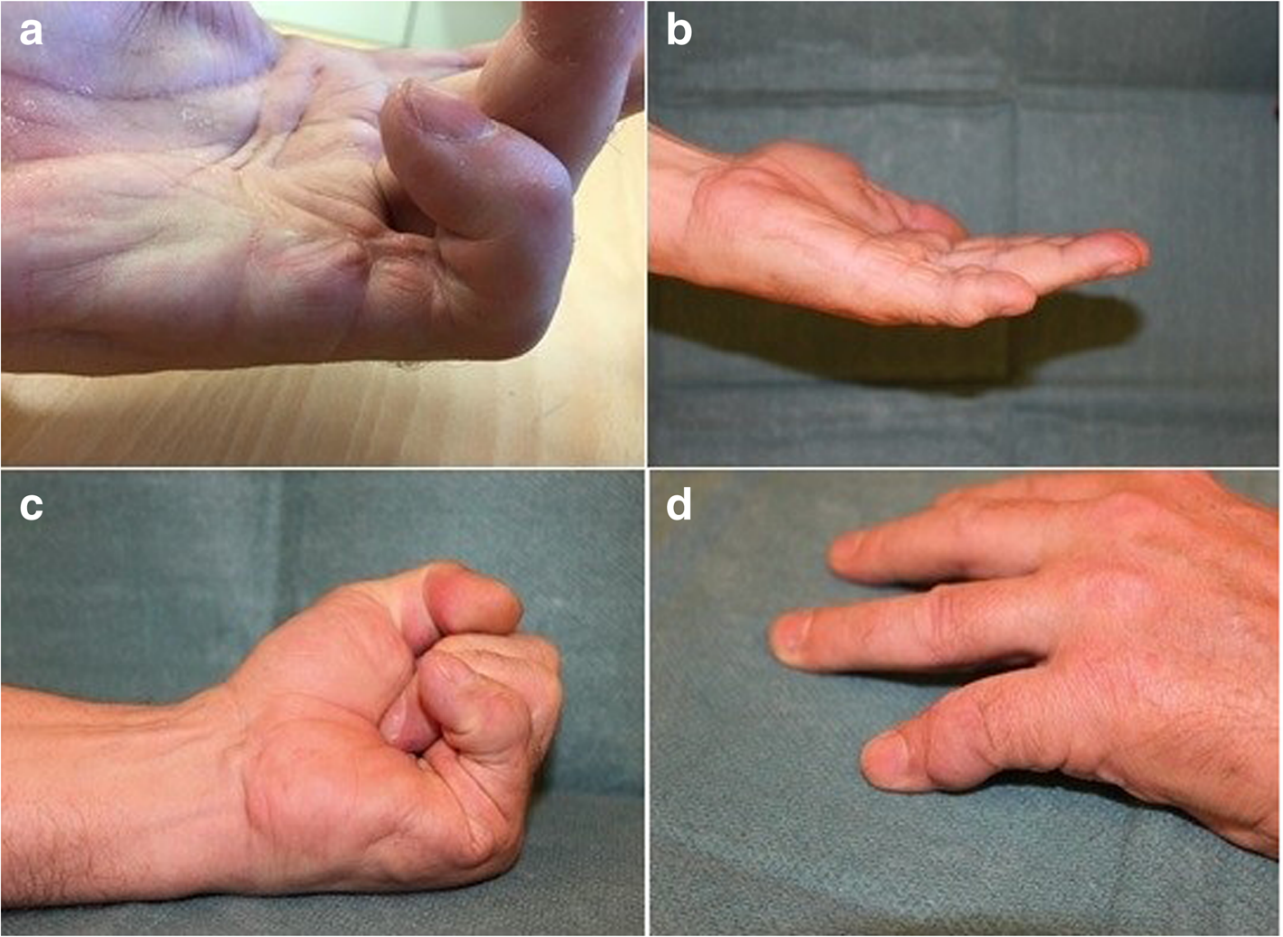
Patient 1 showing (**a**) preoperative status, (**b**) postoperative maximal active extension, (**c**) postoperative maximal active flexion, (**d**) postoperative appearance from dorsal side

**Fig. 2:**
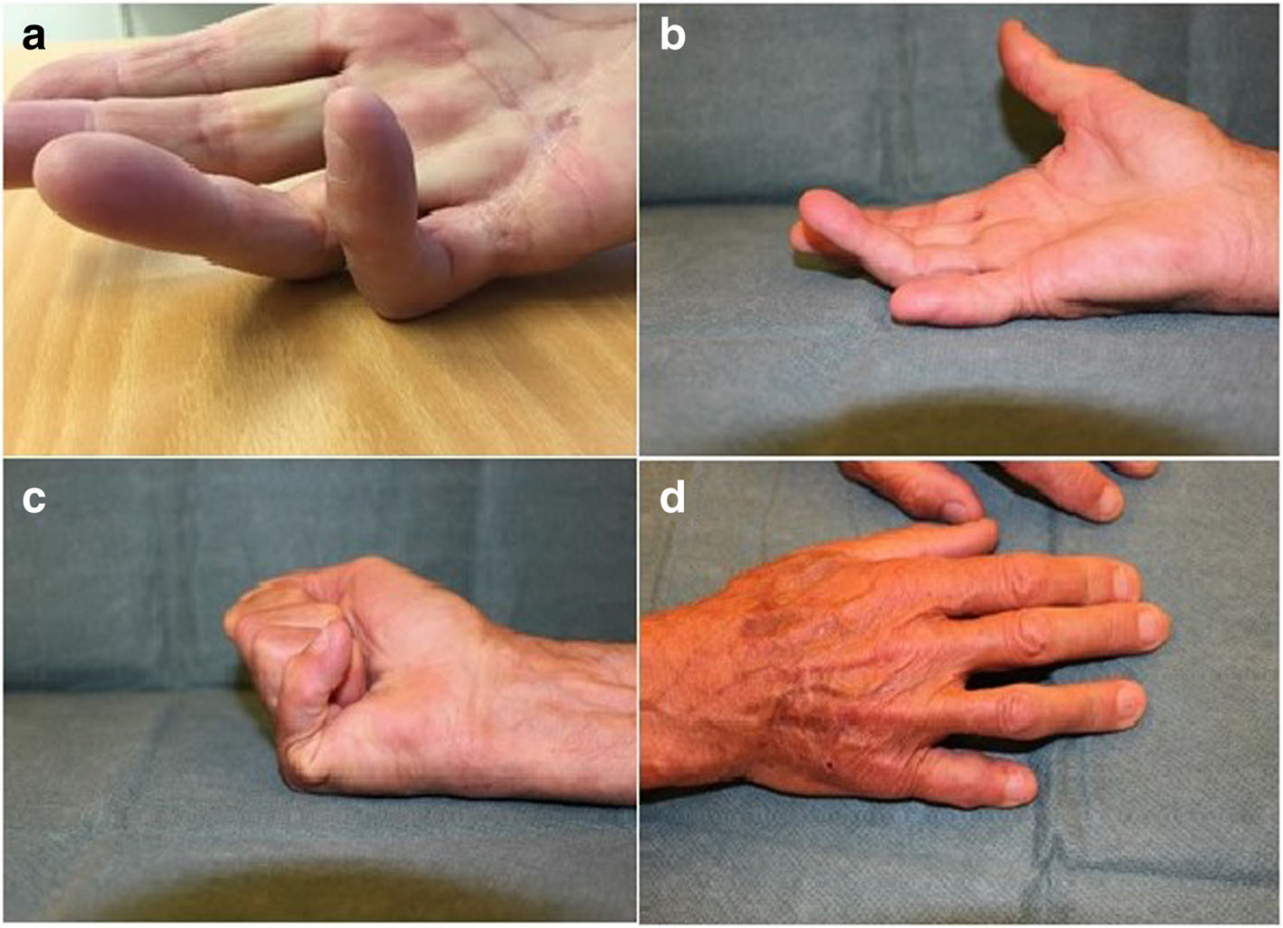
Patient 2 showing (**a**) preoperative status, (**b**) postoperative maximal active extension, (**c**) postoperative maximal active flexion, and (**d**) postoperative appearance from dorsal side

**Fig. 3:**
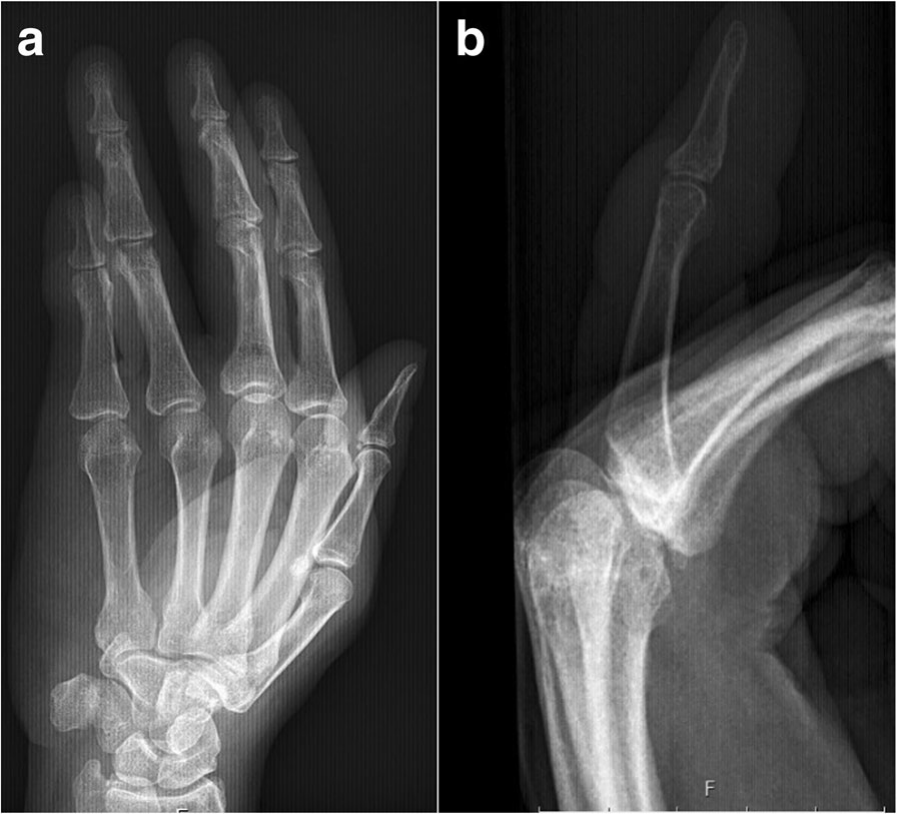
Radiograph of patient 1 showing (**a**) posteroanterior and (**b**) lateral views of the small finger

**Fig. 4:**
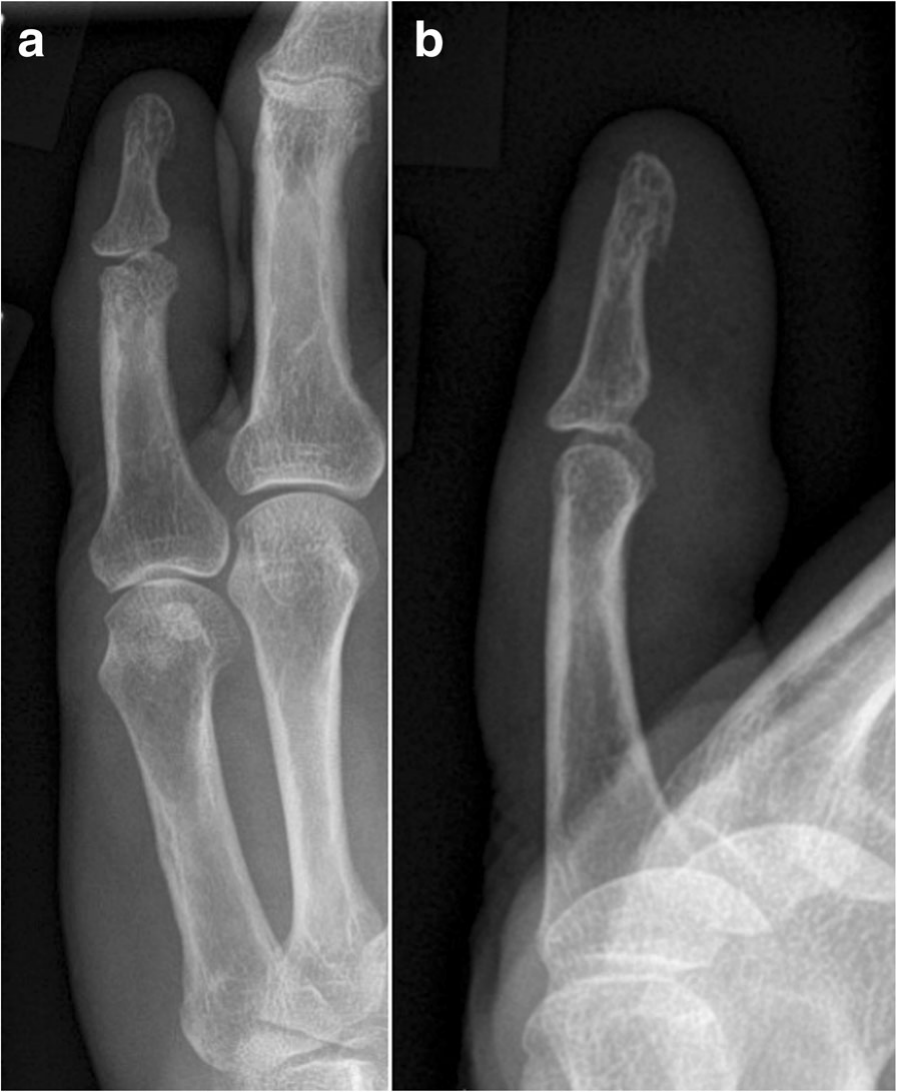
Radiograph of patient 2 showing (**a**) posteroanterior and (**b**) lateral views of the small finger

Both patients regained full extension of the MCP joint and near full extension of the new interphalangeal joint (Table 1, Figs. 1 and 2) with good flexion. The joint was stable on examination. Both patients stated they have no difficulties in performing activities of daily living, no pain and no sensitivity to cold or touch. The second patient was able to resume his hobbies of carpentry, hunting and fishing that were difficult to perform before surgery because of his finger contracture. Both patients are very satisfied with the outcome including the appearance of their hands. No adverse events occurred. The radiographs show congruent joints in both patients (Figs. 3 and 4).

## Discussion

We report a new surgical procedure comprising total excision of the middle phalanx and ligament reconstruction to create a functioning single interphalangeal joint as an alternative to finger amputation in severe recurrent Dupuytren contracture of the small finger. The procedure was used on two patients with good outcome and no adverse events. Both patients are satisfied with the result, and both have requested the same procedure to be carried out on their similarly affected opposite side.

The cosmetic result seems to be acceptable as both patients are satisfied with this aspect and report having received little reaction from their surroundings about the appearance of the hand. This may suggest that despite the shortening of the small finger the hand seems to be perceived as normal by others. Importantly, the patients experience no pain and no cold intolerance and no difficulties in performing daily activities. The first patient has good flexion in the interphalangeal joint but the second patient has limited flexion. However, the flexion deficit does not seem to cause activity limitations.

The two previous case reports of procedures involving middle phalanx excision have reported good results. In the single case of simple excision of the middle phalanx in a patient with recurrent Dupuytren contracture, Teboul et al. [14] reported that good outcome was achieved at 12 months follow-up, with good grip strength (37 kg measured with Jamar, no information is given on contralateral strength), a finger pulp-palm distance of 1 cm, and return to manual work 6 weeks postoperatively. Honecker et al. [13] performed middle phalanx resection with proximodistal fusion in 7 patients, including 4 patients with Dupuytren contracture and reported, at mean follow up was 35 months, significant reduction of pain (from a mean 5.4 preoperatively to 1.4 postoperatively on a 0–10 scale) and improved 11-item disabilities of the arm, shoulder and hand (QuickDASH) score (51 preoperatively to 33 postoperatively). However, 3 of the patients had posttraumatic conditions and one of the patients with Dupuytren’s contracture had rapid recurrence of the disease and bending of the screw-pin. A disadvantage of this procedure is that it results in a completely immobile joint.

This new surgical procedure is technically easy. Postoperative pain and discomfort seems minimal. We have followed the 2 patients for 18 months and 15 months, respectively, and have seen no signs of recurrence but in Dupuytren disease longer follow-up is necessary. In addition longer follow-up can show whether the patients develop symptomatic osteoarthritis in the new interphalangeal joint. The results are encouraging as both patients that had requested amputation because of this debilitating condition have now been free of symptoms and show no worsening after more than a year, which is a longer than was the case in any of their previous treatments.

## Conclusions

This procedure can be a good alternative to amputation in patients with severe recurrent contracture of the small finger PIP joint. Although we have used the procedure only on the small finger it may also be used in severe recurrent contractures involving other fingers in situations when finger amputation is considered. The risk of nerve damage and ensuing phantom pain is reduced and the cosmetic appearance is better than after an amputation.

## Data Availability

All available data are reported in the manuscript; no more data are available.

## Abbreviations

DD: Dupuytren disease
DIP: Distal interphalangeal joint
MCP: Metacarpophalangeal joint
PIP: Proximal interphalangeal joint
